# Applications of Diacylglycerol Acyltransferase for Triacylglycerol Production in *Mortierella alpina*

**DOI:** 10.3390/jof9020219

**Published:** 2023-02-07

**Authors:** Ruilin Yu, Lulu Chang, Jun Cao, Bo Yang, Haiqin Chen, Wei Chen

**Affiliations:** 1 State Key Laboratory of Food Science and Technology, Jiangnan University, Wuxi 214122, China; 2 School of Food Science and Technology, Jiangnan University, Wuxi 214122, China; 3 National Engineering Research Center for Functional Food, Jiangnan University, Wuxi 214122, China

**Keywords:** diacylglycerol acyltransferase, lipidomics, triacylglycerol, linseed oil supplementation, *Mortierella alpina*

## Abstract

Triacylglycerol (TG) with high-value long-chain polyunsaturated fatty acids is beneficial to human health; consequently, there is an urgent need to broaden its sources due to the current growing demand. *Mortierella alpina*, one of the most representative oleaginous fungi, is the only certificated source of dietary arachidonic acid-rich oil supplied in infant formula. This study was conducted to improve TG production in *M. alpina* by homologous overexpression of diacylglycerol acyltransferase (*DGAT*) and linseed oil (LSO) supplementation. Our results showed that the homologous overexpression of *MaDGAT1B* and *MaDGAT2A* strengthened TG biosynthesis and significantly increased the TG content compared to the wild-type by 12.24% and 14.63%, respectively. The supplementation with an LSO concentration of 0.5 g/L elevated the TG content to 83.74% and total lipid yield to 4.26 ± 0.38 g/L in the *M. alpina-MaDGAT2A* overexpression strain. Our findings provide an effective strategy for enhancing TG production and highlight the role of *DGAT* in TG biosynthesis in *M. alpina*.

## 1. Introduction

Triacylglycerol (TG), a type of neutral lipid comprising three fatty acyl moieties, is the major reserve of fatty acids (FAs) and the main form of energy storage in eukaryotes [1]. The FAs attached to a TG usually differ and can be categorized into saturated FAs (SFAs), monounsaturated FAs (MUFAs), and long-chain polyunsaturated FAs (LC-PUFAs), depending on the length of the carbon chain and the number of unsaturated bonds. The different FA compositions of TG lead to a variety of physiological functions, health benefits, and nutritional values. In particular, TG molecules enriched with LC-PUFAs are receiving more research focus owing to their various benefits to human health, which include aiding visual and neurological/brain development [2] and reducing the risk of cancer [3] and cardiovascular disease [4].

The natural sources of TG-carrying LC-PUFAs are plants, oil crop seeds, marine fish, and oleaginous microorganisms that produce lipids over 20% of their dry cell weight (DCW) [5]. Microbial oils have attracted increasing attention because of their abundant and diverse composition of PUFAs and their inexpensive and sustainable access [6]. *Mortierella alpina*, one of the most representative natural producers of high-value LC-PUFAs, has already been used to produce dietary arachidonic acid-rich oil as a supplement in infant formula [7]. The FA composition of *M. alpina* includes palmitic acid (C16:0), stearic acid (C18:0), oleic acid (OA, C18:1), linoleic acid (LA, C18:2), γ-linoleic acid (GLA, C18:3), dihomo-γ-linolenic acid (DGLA, C20:3), and a large amount of arachidonic acid (ARA, C20:4). Another indispensable FA, eicosapentaenoic acid (EPA, C20:5), is usually present in *M. alpina* in trace amounts below 12 °C [8]. Thus, *M. alpina* is an outstanding source of TG molecules with LC-PUFAs.

Diacylglycerol acyltransferase (*DGAT*) catalyzes the final step in TG biosynthesis; it transfers the fatty acyl moiety from fatty acyl-coenzyme A (acyl-CoA) to diacylglycerol (DG) and commits DG to be stored as TG rather than being diverted for phospholipid synthesis [9]. According to the differences in structure and cellular or subcellular localization, *DGAT* is divided into four families: *DGAT1*, *DGAT2*, WS/*DGAT*, and *DGAT3* (CytoDGAT). Among these, two integral membrane-bound enzymes, *DGAT1* and *DGAT2*, are ubiquitously found in eukaryotes without homology [10,11]. In *M. alpina*, the functional characterization and substrate preference of *DGAT1* and *DGAT2A* have been explored in *S. cerevisiae*. *MaDGAT2A* could restore TG biosynthesis in *S. cerevisiae* H1246, and *MaDGAT2B* has also been applied to alter the FA and lipid composition [12,13,14].

There are thousands of lipid molecules in *M. alpina*, and their metabolism is connected through numerous pathways and networks. Lipidomics enables large-scale qualitative and quantitative analyses of these lipid molecules to reveal their complex profiles. The total lipid derived from *M. alpina* comprises five lipid classes, including glycerolipids, glycerophospholipids, sphingolipids, FAs, and sterols [15]. The major lipid subclasses in *M. alpina* are TG, DG, phosphatidylcholine (PC), and phosphatidylethanolamine (PE), which account for more than 95% of its total lipid content [16]. Benefiting from the rapid development of analytical instruments and technologies, liquid chromatography and mass spectrometry assist in the accurate separation and identification of lipid molecules during lipidomics analysis [17]. Furthermore, ultra-performance liquid chromatography (UPLC) coupled with a Q Exactive Orbitrap mass spectrometer (QE-MS/MS), which provides ultrahigh resolution and mass accuracy, has been broadly applied for lipidomics analysis [18,19,20].

In previous studies, the overexpression of *DGAT* has been applied to increase TG content and to accumulate desired FAs in several species, such as yeast [21,22] and algae [23,24]. *DGAT* is one of the most effective enzymes for enhancing lipid accumulation due to its key role in TG biosynthesis. Supplementation with linseed oil (LSO) is an inexpensive and effective strategy for increasing the yield of high-value LC-PUFAs in *M. alpina* [25]. However, the effects of *M. alpina* *DGAT* on TG biosynthesis in vivo and the application of LSO supplementation on TG accumulation have not yet been explored.

In this study, four identified genes, *MaDGAT1A*, *MaDGAT1B*, *MaDGAT2A*, and *MaDGAT2B*, were overexpressed, and the effects of *MaDGAT1B* and *MaDGAT2A* on lipid profiles were investigated. Furthermore, LSO was administered to the *M. alpina-MaDGAT2A* overexpression and wild-type strains during fermentation. Subsequently, the total biomass of the mycelium was measured, and its lipid composition was analyzed to explore the effects of LSO supplementation on lipid biosynthesis. Thus, in this study, we attempted to provide a new perspective for obtaining more TG molecules with high-value LC-PUFAs for production applications.

## 2. Materials and Methods

### 2.1. Strains and Plasmids

*M. alpina* ATCC 32222 was purchased from American Type Culture Collection (Manassas, VA, USA). The uracil-auxotrophic strain *M. alpina* CCFM 501 was constructed by homologous recombination to knock out the *ura5* gene encoding orotate phosphoribosyl transferase (OPRTase, EC.2.4.2.10), based on the wild-type strain *M. alpina* ATCC 32222. The binary expression vector pBIG2-ura5s-ITs was constructed in a previous study and conserved in the *E. coli* top 10 strain [26]. *Agrobacterium tumefaciens* CCFM 834 was a transfer DNA (T-DNA) donor for *A. tumefaciens*-mediated transformation. The uracil-auxotrophic strain, *M. alpina* CCFM 501, was used as the transformation recipient to construct a recombinant strain. CCFM refers to the Culture Collection of Food Microorganisms of Jiangnan University, School of Food Science and Technology, Research Center of Food Biotechnology.

### 2.2. Sequence Alignment, Cloning, and Construction of Homologous Overexpression Vector for MaDGATs of M. alpina

Using several identified cDNA sequences of *DGAT1* and *DGAT2* from different species (*Homo sapiens*, *Mus musculus*, *Arabidopsis thaliana*, *P. tricornutum*, and *Paracoccidioides brasiliensis*) listed in the National Center for Biotechnology Information (NCBI; https://www.ncbi.nlm.nih.gov/ (accessed on 30 January 2023)) as queries, the coding sequences of *M. alpina DGAT1* and *DGAT2* were obtained by homologous alignment in the genome database of *M. alpina* ATCC 32222 [15].

Total RNA was extracted from *M. alpina* using the Trizol method and reverse transcribed to obtain cDNA. The target gene fragment was amplified by PCR, using cDNA as a template and KOD high-fidelity enzyme as an extension enzyme. The fragment was then ligated downstream of the His550 promoter, which was modified from the promoter of H4.1 genes from histone H4 isolated from *M. alpina*, in the vector pBIG2-ura5s-ITs and transferred into *Agrobacterium tumefaciens* by electroporation (2.5 kV, 5.0 ms).

### 2.3. Culture Conditions of M. alpina

*M. alpina* was cultured in a glucose yeast extract (GY) agar slant (20 g/L glucose, 10 g/L yeast extract, 2 g/L KNO_3_, 1 g/L NaH_2_PO_4_, 3 g/L MgSO_4_·7H_2_O, 20 g/L agar, and 0.1 g/L uracil (for uracil auxotroph strain)) at 4 °C for preservation and spore production. Spores were collected with normal saline, filtered through Miracloth (Calbiochem, Germany), and centrifuged at 12,000× *g* for 25 min at 4 °C. *M. alpina* mycelium was transferred from the GY agar slant into 100 mL of broth medium (20 g/L glucose, 5 g/L yeast extract, 10 g/L KNO_3_, 1 g/L KH_2_PO_4_, 0.25 g/L MgSO_4_·7H_2_O, and 0.1 g/L uracil (for uracil auxotroph strain)) in a 250 mL flask and shaken for 48 h at 200 r/min and 28 °C. The mycelium pellets in suspension were dispersed using basic ULTRA-TURRAX (IKA, Germany) and transferred into a new broth medium (inoculated dose of 1%, *v*/*v*). This step was repeated for three generations, each for 48 h. The dispersed mycelial suspension of the last generation was inoculated in the broth medium (with 30 g/L glucose) in a fermentation culture for 168 h at 200 r/min and 28 °C.

### 2.4. A. Tumefaciens-Mediated Transformation

*A. tumefaciens*, which had been transferred into the corresponding vectors, was streaked onto yeast extract peptone (YEP) agar medium. The single colony was inoculated into YEP medium with 100 μg/mL kanamycin and 100 μg/mL rifampicin at 30 °C for 36 h away from light until the medium turned orange; it was then transferred into a minimal medium (MM) with 7.8 g/L 2-(*N*-Morpholino)ethanesulfonic acid (MES) (inoculated dose was 1%, *v*/*v*) at 28 °C for 36 h until the OD_660_ reached 1.5. Subsequently, *A. tumefaciens* was diluted and cultured in an induction medium (IM) containing 100 μg/mL acetosyringone and 7.8 g/L MES at an initial OD_660_ of 0.2 away from light at 30 °C for 12 h until the OD_660_ reached 0.8.

The spores of the uracil-auxotrophic *M. alpina* strain CCFM501 were collected approximately 12 h before the transfer of *A. tumefaciens* to the IM, as described above. The well-grown *A. tumefaciens* in IM was serially diluted to an OD_660_ of 0.2, 0.4, and 0.6 and mixed with 10^7−8^ spores/mL of uracil-auxotrophic *M. alpina* spore suspension. Next, the mixture was coated onto induction agar medium, which was covered with cellophane membranes and supplemented with 0.1 g/L uracil for co-cultivation away from light for 24 h at 16 °C and, then, for 24 h at 28 °C. Subsequently, the cellophane membranes were transferred to an uracil-free synthetic complete (SC) agar medium with 100 μg/mL cefotaxime and 100 μg/mL spectinomycin for screening after the appropriate density of mycelium and bacteria had grown on the membrane. Mycelia from the fungal colonies were picked and transferred to new uracil-free SC agar plates one by one. This step was repeated thrice to obtain genetically stable transformants.

### 2.5. Extraction of Genomic DNA and Identification of Transformants

A Biospin fungal genomic DNA extraction kit (Bioer Technology, Hangzhou, China) was used to extract genomic DNA from *M. alpina* according to the manufacturer’s instructions. As previously described, the presence of T-DNA in the genome of transformants was identified using the universal primers Hispro F1 and TrpC R1. The PCR product was collected by gel extraction and purified using a DNA purification kit (Thermo Scientific, Waltham, MA, USA) for sequencing.

### 2.6. Biomass Analysis, Lipid Extraction, and Targeted Analysis of FA Profiles

After 168 h of fermentation, the fresh mycelia were obtained by fast filtration using a paper filter, washed with distilled water to remove the culture medium, and frozen at −80 °C. Then, they were dried in a vacuum freeze-dryer overnight to quantify the DCW. The freeze-dried biomass was ground into a fine powder for total lipid extraction. All the lipid types were hydrolyzed with 4 mol/L hydrochloric acid solution, extracted with methanol:chloroform (1:2, *v*/*v*) from approximately 30 mg of powder, and methyl-esterified with 4% (*v*/*v*) H_2_SO_4_ in methanol for further analysis. The FA profiles and total lipid yield were analyzed in the form of FA methyl ester using gas chromatography-mass spectrometry (GC-MS) analysis (GCMS-QP2010 Ultra, Shimadzu, Japan) with a DB-WAXetr column (30 m × 0.32 mm; film thickness, 0.25 μm). Pentadecanoic acid (C15:0) was used as the internal standard for relative quantification, and the temperature program was performed as previously described [15,16].

### 2.7. Untargeted Lipidomics Analysis

To reduce contamination of the residual cell contents, a modified methyl tert-butyl ether (MTBE) extraction method was used for lipid extraction for untargeted lipidomics analysis [27,28]. Briefly, 10 mg of the ground mycelial pellet was weighed and placed in a 1.5 mL centrifuge tube. The mycelia were mechanically broken using a homogenizer after adding 225 μL of cold methanol and Zirconia/Silica Beads (0.5 mm). Next, 750 μL of cold MTBE was added and vortexed for 3 min at 2000 rpm; then, 187.5 μL of MS-grade water was added to induce phase separation. Subsequently, the samples were vortexed and centrifuged at 4 °C and 12,000× *g* for 15 min; the upper phase was collected in a new tube, and the lower phase was re-extracted using cold MTBE. The combined organic phases were then dried using a vacuum centrifuge. The extracted lipids were reconstituted in 200 μL of CHCl_3_/methanol/water (60:30:4.5, *v*/*v*/*v*) for further analysis.

Lipidomics analysis was performed using a Dionex UltiMate 3000 UPLC system (Santa Clara, CA, USA) coupled with a Q Exactive Orbitrap mass spectrometer (Thermo Fisher, Sunnyvale, CA, USA). The lipid extract was separated on a Waters Acquity UPLC BEH C18 column (100 × 2.1 mm; film thickness, 1.7 μm) maintained at 40 °C. We used a binary solvent system comprising mobile phases A (60:40 acetonitrile/water solution containing 10 mM ammonium formate and 0.1% formic acid) and B (90:10 isopropanol/acetonitrile containing 10 mM ammonium formate and 0.1% formic acid). The elution gradient was as follows: in the positive ion mode, 0.0–3.0 min, 0–70% B; 3.0–26.0 min, 70% B increasing linearly to 90%; 26.0–27.0 min, 90% B; 27.0–28.0 min, 90% B decreasing linearly to 0%; and 28.0–30.0 min, 0% B; in the negative ion mode, 0.0–3.0 min, 10–70% B; 3.0–12.0 min, 70% B increasing linearly to 80%; 12.0–15.0 min, 80–95% B; 15.0–15.1 min, 95% B decreasing linearly to 10%; and 15.1–18.0 min, 10% B. The mobile phase flow rate was 0.25 mL/min, the autosampler temperature was 15 °C, and the injection volume was 2 μL.

The MS1 and MS2 data were acquired using a Q-Exactive Orbitrap mass spectrometer through data-dependent acquisition in the range of 200–1200 for both the positive and negative ion modes. The detailed parameters were set as previously described, including lipid identification and alignment [16]. Lipid identification and alignment were performed using LipidSearch (version 4.1, Thermo Fisher Scientific, Waltham, MA, USA). The product search type was performed with precursor tolerance (±5 ppm) and product tolerance (±5 ppm). Lipids with m scores of <5, a peak area of <1 × 10^5^, and a relative standard deviation (SD) of >30% were excluded. The ID quality filters were selected as grades A and B. The adduct ions were +H and +NH_4_ in the positive ion mode and −H, +HCOO, and −2H in the negative ion mode. The content of a particular lipid class is obtained by dividing the total peak area of that lipid class by the total peak area of all lipid molecules.

### 2.8. Statistical Analysis

All data are presented as means ± SD, representing at least three independent experiments. SPSS software version 20.0 (IBM Corp., Armonk, NY, USA) and GraphPad Prism version 8 (GraphPad Software, San Diego, CA, USA) were used to perform statistical analysis and visualization. Paired two-tailed, two-tests were used for two-group comparisons, and one-way analysis of variance with Tukey’s test was used for multiple comparisons. Statistical significance was defined as *p* < 0.05.

## 3. Results

### 3.1. Identification and Screening of the M. alpina-MaDGAT1A/1B/2A/2B Recombinant Strains 

**Figure 1:**
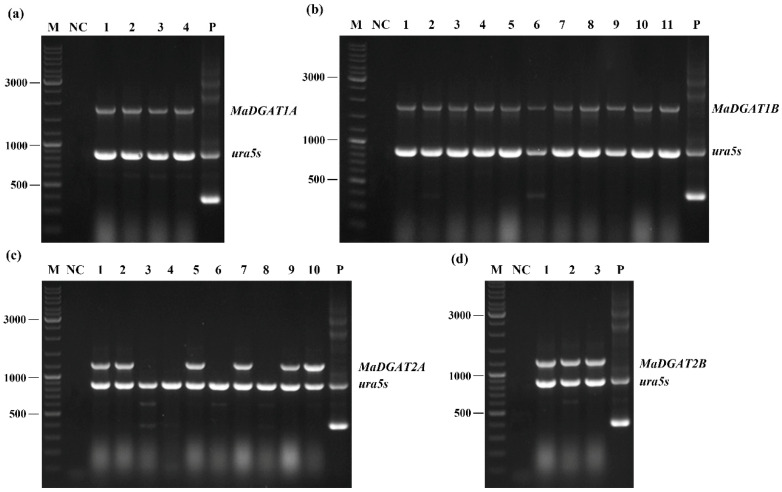
PCR identification of *M. alpina-MaDGAT1A*/*1B*/*2A*/*2B* recombinant strains. The lanes are depicted as follows: (**a**) 1–4, four *M. alpina-MaDGAT1A* recombinant strains; (**b**) 1–11, 11 *M. alpina-MaDGAT1B* recombinant strains; (**c**) 1–10, 10 *M. alpina-MaDGAT2A* recombinant strains; (**d**) 1–3, three *M. alpina-MaDGAT2B* recombinant strains; M, marker; NC, negative control (ddH_2_O); P, plasmid pBIG2-ura5s-ITs. The length of the PCR products of *ura5s* and ITs are 818 bp and 352 bp, respectively.

The *M. alpina DGAT1A*, *DGAT1B*, *DGAT2A*, and *DGAT2B* genes were obtained from the genome of *M. alpina* ATCC 32222 based on the results of the sequence alignment and ligated to the binary expression vector pBIG2-ura5s-ITs. The T-DNA regions containing *MaDGAT1A*, *MaDGAT1B*, *MaDGAT2A*, and *MaDGAT2B*, with the selection marker *urs5s*, were inserted into the genome of *M. alpina* CCFM 501 by *A. tumefaciens*-mediated transformation. Several transformants with genetic stability were successfully obtained, and segments of *MaDGAT1A*, *MaDGAT1B MaDGAT2A*, and *MaDGAT2B* were amplified from the genome of the transformants by PCR. Figure 1 shows that the presence of the corresponding gene segments was detected in all transformants except in *M. alpina-MaDGAT2A*-3/4/6/8.

**Figure 2:**
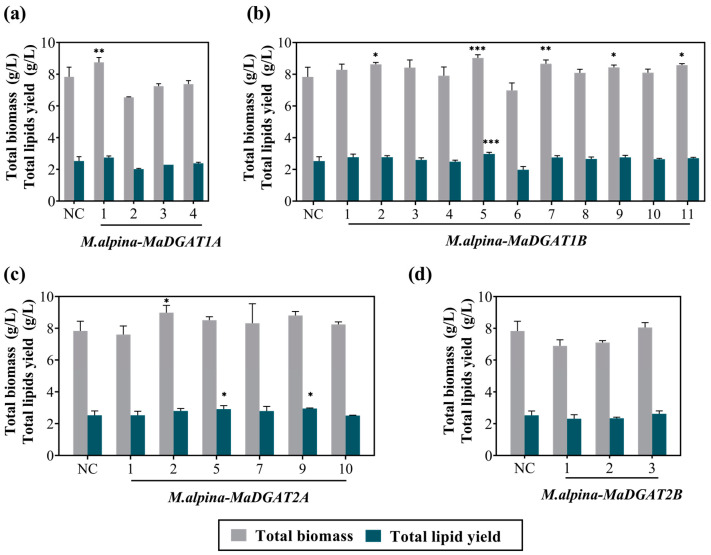
Total biomass and fatty acid yield of *M. alpina*-*MaDGAT1A*/*1B*/*2A*/*2B* recombinant strains. (**a**) *M. alpina*-*MaDGAT1A*, (**b**) *M. alpina*-*MaDGAT1B*, (**c**) *M. alpina*-*MaDGAT2A*, and (**d**) *M. alpina*-*MaDGAT2B*; NC, negative control. The total lipid yield was calculated by multiplying the total lipid content of dry cell weight by the total biomass. The significant differences between NC and each *DGAT* recombinant strain are indicated by * *p* < 0.05, ** *p* < 0.01, and *** *p* < 0.001. For each group, three biological replicates were used.

Subsequently, we analyzed the growth and total lipid yield of the above transformants, including four *M. alpina-MaDGAT1A* recombinant strains, eleven *M. alpina-MaDGAT1B* recombinant strains, six *M. alpina-MaDGAT2A* recombinant strains, and three *M. alpina-MaDGAT2B* recombinant strains. The complementary-phenotype strain obtained by transforming the vector pBIG2-ura5s-ITs into the uracil-auxotrophic strain was used as the negative control (Figure S1). After 7 d of fermentation in the broth medium with 30 g/L glucose, compared to the control group, no adverse changes in growth and lipid production were observed in the above verified transformants, except for a decrease in the total lipid yield of *M. alpina-MaDGAT1B*-6. In terms of biomass accumulation, *M. alpina-MaDGAT1A*-1 (Figure 2a), *M. alpina-MaDGAT1B*-2/5/7/9/11 (Figure 2b), and *M. alpina-MaDGAT2A*-2 (Figure 2c) showed remarkable improvements. No significant improvement was observed in the three *M. alpina-MaDGAT2B* recombinant strains (Figure 2d). Interestingly, the total lipid yield of *M. alpina-MaDGAT1B*-5 and *M. alpina-MaDGAT2A*-9 increased by 17.85% and 17.06%, respectively, compared with the control group, indicating that these two strains possessed higher lipid accumulation abilities than all the other recombinant strains.

### 3.2. Lipidomics Profiles of M. alpina-MaDGAT1B and M. alpina-MaDGAT2A

Based on the above results, *M. alpina-MaDGAT1B*-5 and *M. alpina-MaDGAT2A*-9 were selected for further analysis. We investigated the effects of *MaDGAT1B* and *MaDGAT2A* overexpression on different lipid compositions of *M. alpina*. After fermentation in broth medium (with 30 g/L glucose) for 7 d, we separated and identified the lipids of *M. alpina-MaDGAT1B*, *M. alpina-MaDGAT2A*, and *M. alpina* ATCC 32222 by UPLC-QE-MS/MS, and the repeatability and reliability of the data were confirmed by principal component analysis (Figure S2). Subsequently, we determined the biomass of the mycelium with its total lipid yield and analyzed its FA profile by GC-MS. Although the biomass of the *M. alpina-MaDGAT1B*/*2A* overexpression strains showed a slight decline, the total lipid yield of *M. alpina-MaDGAT1B*/*2A* increased considerably compared to the wild-type strain, up to 3.66 g/L and 3.68 g/L, respectively (Table 1).

**Figure 3:**
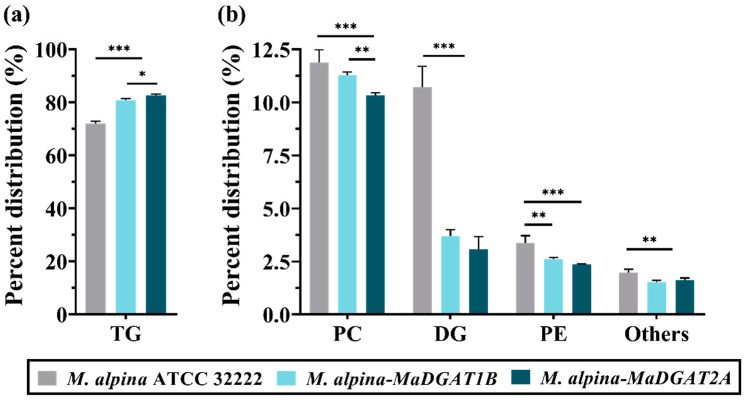
Major lipid content in *M. alpina* ATCC 32222, *M. alpina-MaDGAT1B*, and *M. alpina-MaDGAT2A*. (**a**) The content of TG and (**b**) PC, DG, PE, and other lipids. The significant differences are indicated by * *p* < 0.05, ** *p* < 0.01, and *** *p* < 0.001. For each group, three biological replicates were used. TG, triacylglycerol; PC, phosphatidylcholine; DG, diacylglycerol; PE, phosphatidylethanolamine.

Remarkably, the TG content in *M. alpina-MaDGAT1B*/*2A* accounted for 80.86% and 82.58% of the total lipid, respectively (Figure 3a), which were significantly higher than that of *M. alpina* ATCC 32222 (72.04%), increased by 12.24% and 14.63%, respectively. The DG content in *M. alpina-MaDGAT1B*/*2A* decreased from 10.71% to 3.70% and 3.08%, respectively, compared with that in *M. alpina* ATCC 32222 (Figure 3b). Meanwhile, the PC content in *M. alpina-MaDGAT1B*/*2A* decreased from 11.88% (wild-type strain) to 11.28% and 10.33%, and the PE content decreased from 3.37% to 2.62% and 2.37%, respectively (Figure 3b). The content of other lipids in the *MaDGAT1B* and *MaDGAT2A* overexpression strains also showed a decreasing trend.

**Figure 4:**
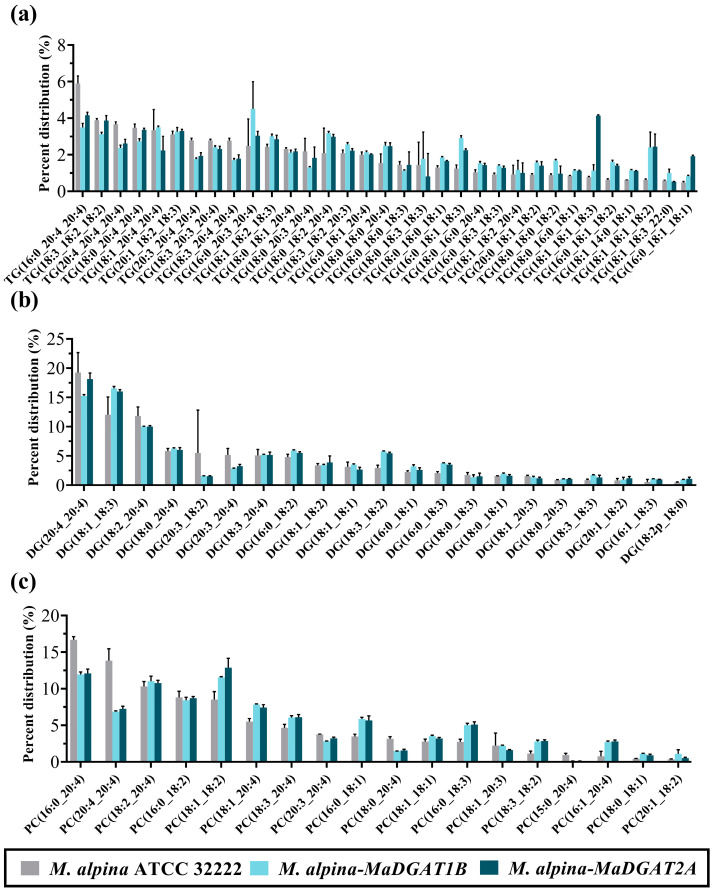
Lipidomics profiles in *M. alpina* ATCC 32222, *M. alpina-MaDGAT1B*, and *M. alpina-MaDGAT2A*. (**a**) The distribution of TG profiles, (**b**) DG profiles, and (**c**) PC profiles. For each group, three biological replicates were used.

Subsequently, we focused on the composition of the fatty acyl moiety in TG molecules. The TG molecules containing more than 1% of the total TG are shown in Figure 4a. In *M. alpina* ATCC 32222, the prominent TG molecules were TG (16:0_20:4_20:4), TG (20:4_20:4_20:4), TG (20:3_20:4_20:4), and TG (18:3_20:4_20:4); however, the content of these TG molecules were reduced in *M. alpina-MaDGAT1B*/*2A* compared to wild-type strain. In contrast, TG (16:0_20:3_20:4), TG (16:0_18:1_18:3), and TG (18:0_18:0_20:4) in *M. alpina-MaDGAT1B* and TG (18:1_18:1_18:3), TG (18:1_18:1_18:2), and TG (16:0_18:1_18:1) in *M. alpina-MaDGAT2A* exceeded their content in the wild-type strain. The species of major DG and PC molecules were much less than those of the TG molecules (Figure 4b). The percentage of DG molecules containing C20:4, such as DG (20:4_20:4) and DG (18:2_20:4), was higher in the wild-type than that in *M. alpina-MaDGAT1B*/*2A*, whereas the reverse was found for DG molecules containing C18 FAs, such as DG (18:1_18:3) and DG (18:3_18:2). A similar situation was observed with the PC molecules, where the percentages of PC (20:4_20:4) and PC (16:0_20:4) were higher in the wild-type than those in *M. alpina-MaDGAT1B*/*2A*, but the percentages of PC (18:1_18:2) and PC (16:0_18:1) were higher in *M. alpina-MaDGAT1B*/*2A* than those in the wild-type (Figure 4c).

### 3.3. Effect of LSO Supplementation on TG Production of M. alpina-MaDGAT2A

*M. alpina-MaDGAT2A* had the highest TG content and was chosen to explore the effects of exogenous LSO supplementation on *MaDGAT2A* and the resulting TG accumulation. On the third day of fermentation, LSO was added to the broth medium (with 30 g/L glucose) until the concentration reached 0.5 g/L.

**Figure 5:**
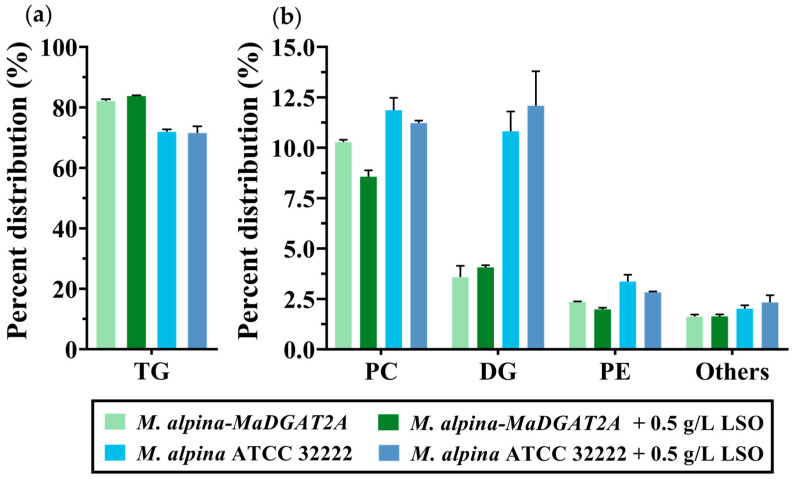
Major lipid content in *M. alpina* ATCC 32222 and *M. alpina-MaDGAT2A* with or without LSO supplementation. (**a**) The content of TG and (**b**) PC, DG, PE, and other lipids. For each group, three biological replicates were used.

As shown in Table 2, the total biomass and total lipid yield increased with LSO supplementation in both strains, and total lipid yield of *M. alpina-MaDGAT2A* reached 4.26 ± 0.38 g/L. Interestingly, the increase in total biomass and total lipid yield in *M. alpina-MaDGAT2A* were greater than those in the wild-type strain. Moreover, the TG content reached 83.74% in *M. alpina-MaDGAT2A* with 0.5 g/L LSO supplementation; however, the TG content of the wild-type strain showed a slight decline from 71.93% to 71.53% (Figure 5a). The PC content of both groups showed different levels of reduction, while the increase in DG content in *M. alpina-MaDGAT2A* was much lower than that in the wild-type strain (Figure 5b). The FA profiles of two strains with LSO supplementation were shown in Table S2. 

## 4. Discussion

In this study, we successfully overexpressed four *M. alpina DGAT* genes in vivo with the help of *A. tumefaciens* and analyzed lipid profiles of *M. alpina-MaDGAT1B*/*2A* using UPLC-QE-MS/MS to explore the potential of *MaDGATs* in TG production. The overexpression of *MaDGAT1B* and *MaDGAT2A* significantly elevated TG accumulation. Furthermore, supplementation with LSO improved the mycelial biomass and TG content. Our findings provide new ideas for enhancing TG production.

*DGAT* catalyzes the last and most critical step of TG biosynthesis in the Kennedy pathway. In the current study, comparing total biomass and total lipid yield of overexpression strains with complementary-phenotype and wild-type strain showed that the overexpression of *MaDGATs* improves lipid accumulation. In the meantime, the transformants of each *MaDGAT* showed different performances in terms of total biomass and total lipid yield, which may be due to the different insertion sites of T-DNA during transformation [26,29]. The lipid accumulation in *M. alpina* is positively correlated with TG content [16]. Thus, we selected transformants that were more superior in total lipid yield than the control group for further exploration and application in TG biosynthesis. The promotion of *DGAT* on lipid accumulation has been reported in many previous studies. The homologous overexpression of *McDGAT2d* gave an improvement to lipid accumulation under static solid fermentation [30], and the overexpression of endogenous *PtDGAT1* increased by more than double in TG and total lipid compared to that of the wild-type *Phaeodactylum tricornutum*, which reached 57% and 73% of dry weight, respectively [31]. In addition, the overexpression of endogenous *PtDGAT2B* resulted in enhancing the neutral lipid content by 69% [32].

In a previous study, we performed a lipid analysis of *M. alpina* using a TSQ triple quadrupole tandem mass spectrometer, including glycerolipid, glycerophospholipid, sterol lipid, and sphingolipid analyses [15]; however, the identification of the fatty acyl moiety in TG molecules could not be performed accurately. In this study, we identified TG molecules using an advanced Q Exactive Orbitrap mass spectrometer. The lipidomics analysis revealed more C18 FAs than C20 FAs in the TG profiles of *M. alpina-DGAT1B*/*2A* than in those of wild-type strain, which is also consistent with the FA profiles (Table S1). These results corroborate the previous findings that *MaDGAT2A* prefers LA and GLA over ARA with mixtures of *n*-6 PUFA (LA, GLA, DGLA, and ARA) supplementation in recombinant yeast [12].

Furthermore, comparing the TG content of *M. alpina-MaDGAT1B* and *M. alpina-MaDGAT2A* with that of wild-type demonstrated that *MaDGAT1B* and *MaDGAT2A* could significantly promote the conversion of DG to TG but not the synthesis of phospholipids, where the content of PE and PC decreased (Figure 3). Another important finding was that the overexpression of *MaDGAT1B* and *MaDGAT2A* enhanced the total lipid yield, regardless of a slight decrease in biomass, compared to the wild-type strain (Table 1). It is worth noting that these improvements in lipid and TG accumulation were more significant in *M. alpina-MaDGAT2A*, indicating that *MaDGAT2A* provides more power to produce TG and that *MaDGATs* may play different roles in TG biosynthesis. The different substrate preferences of *MaDGATs* lead to their different effects on TG synthesis. *NoDGAT1A* knockdown and overexpression appreciably impacted TG accumulation in *N. oceanica*; hence, the overexpression of *NoDGAT1A* increased TG yield by 47% [33]. On the other hand, *NoDGAT2s* that possessed distinct substrate specificity were used to produce TG molecules with tailored LC-PUFAs [34]. The results of the heterologous expression in yeast, in vitro assay, and homologous overexpression all revealed that *PtDGAT1* is superior to the other five *PtDGATs* in biosynthesizing TG [31]. In summary, these results show that *MaDGAT* overexpression is a highly effective metabolic engineering strategy to increase both total lipid and TG production.

After supplementation with LSO, the increase in the total lipid yield of *M. alpina-MaDGAT2A* was greater than that of the wild-type (Table 2). The TG content also showed an increase in *M. alpina-MaDGAT2A*, and the increase in DG content in the wild-type strain was greater than that in *M. alpina-MaDGAT2A* (Figure 5). However, the TG content did not increase significantly with the increase in LSO concentration (Figure S3). These findings suggest that *MaDGAT2A* can enhance lipid production and TG biosynthesis with LSO supplementation more than that in the absence of LSO, but the wild-type strain may have difficulty converting such a lot of LSO. Peony seed oil was supplied to *M. alpina* in previous study, and the EPA production was improved to 588.5 ± 29.6 mg/L in a 5-L bioreactor with a *Micromonas pusilla* delta-6 desaturase gene overexpressed, which resulted in a 26.2-fold increase compared to EPA production in wild-type *M. alpina* [35]. Nevertheless, LSO supplementation with *MaDGAT2A* overexpression strain did not obtain such a great boost. A possible explanation for this result may be that the high intrinsic TG content restricts enhancement range.

Overall, our findings revealed the potential and high application value of *M. alpina* *DGATs* in enhancing TG production and lipid accumulation. With LSO supplementation, TG production by *M. alpina-MaDGAT2A* could be further enhanced by increasing substrate availability. Our investigation offers new strategies for improving TG production from the perspective of metabolic engineering and substrate supplementation while also supporting industrial TG production with LC-PUFAs. In future work, rewriting the carbon flux into FA synthesis to provide sufficient substrate for TG synthesis and blocking TG breakdown pathways by multi-gene operation system are potential strategies to further increase LC-PUFA and TG production. Furthermore, differences in the substrate preference of *MaDGATs* could be exploited to biosynthesize TG molecules with tailored LC-PUFAs.

## Figures and Tables

**Table 1:** Total biomass and fatty acid yield of *M. alpina-MaDGAT1B*, *M. alpina-MaDGAT2A*, and *M. alpina* ATCC 32222. For each group, three biological replicates were used.

Strains	Total Biomass (g/L)	Total Lipid Yield (g/L)
*M. alpina* ATCC 32222	10.35 ± 0.47	3.42 ± 0.34
*M. alpina-MaDGAT1B*	9.11 ± 0.12	3.66 ± 0.1
*M. alpina-MaDGAT2A*	9.39 ± 0.26	3.69 ± 0.25

**Table 2:** Total biomass and total lipid yield of *M. alpina* ATCC 32222 and *M. alpina-MaDGAT2A* without LSO and with 0.5 g/L LSO supplementation. For each group, three biological replicates were used. LSO, linseed oil.

Strains	LSO Concentration (g/L)	Total Biomass (g/L)	Total Lipid Yield (g/L)
*M. alpina* ATCC 32222	0	10.35 ± 0.47	3.42 ± 0.34
	0.5	10.82 ± 0.1	3.71 ± 0.11
*M. alpina-MaDGAT2A*	0	9.39 ± 0.26	3.69 ± 0.25
	0.5	9.99 ± 0.19	4.26 ± 0.38

## Data Availability

The datasets that support the findings of current study are available from the corresponding author upon reasonable request.

## Supplementary Materials

The following supporting information can be downloaded at https://www.mdpi.com/article/10.3390/jof9020219/s1, Figure S1. PCR identification of *M. alpina-pBIG2-ura5s* complementary-phenotype strains; Figure S2. PCA score plot showing the discrimination between each group; Figure S3. The effect of different concentrations of LSO on *M. alpina-MaDGAT2A* and *M. alpina* ATCC 32222. (a), Total biomass and total lipid yield of *M. alpina-MaDGAT2A* and *M. alpina* ATCC 32222. (b), content of major lipids with different concentrations of LSO in M. *alpina-MaDGAT2A* and *M. alpina* ATCC 32222; Table S1. Fatty acid profiles of *M. alpina-MaDGAT1B*, *M. alpina-MaDGAT2A*, and *M. alpina* ATCC 32222; Table S2. Fatty acid profiles of *M. alpina-MaDGAT2A* and *M. alpina* ATCC 32222 with different concentrations of linseed oil.
