# Supplementary material for: Applications of Diacylglycerol Acyltransferase for Triacylglycerol Production in Mortierella alpina
Source: J Fungi (Basel). 2023 Feb 7;9(2):219. doi: 10.3390/jof9020219 (PMC9965251; doi:10.3390/jof9020219)
Supplement: Supplementary file 1 [file jof-09-00219-s001.zip › jof-2166621-supplementary.pdf]

Supporting Information

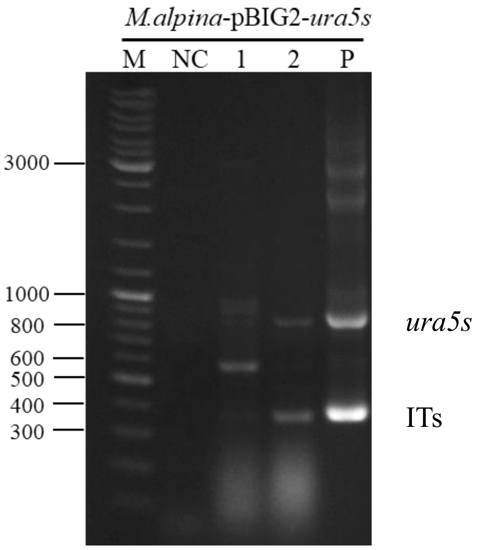

Figure S1. PCR identification of *M. alpina-pBIG2-ura5s* complementary-phenotype strains. The lanes are depicted as follows: 1-2, two *M. alpina*-pBIG2-*ura5s* recombinant strains; M, marker; NC, negative control (ddH_2O); P, plasmid pBIG2-ura5s-ITs. The length of PCR products of ura5s and ITs are 818bp and 352bp, respectively.

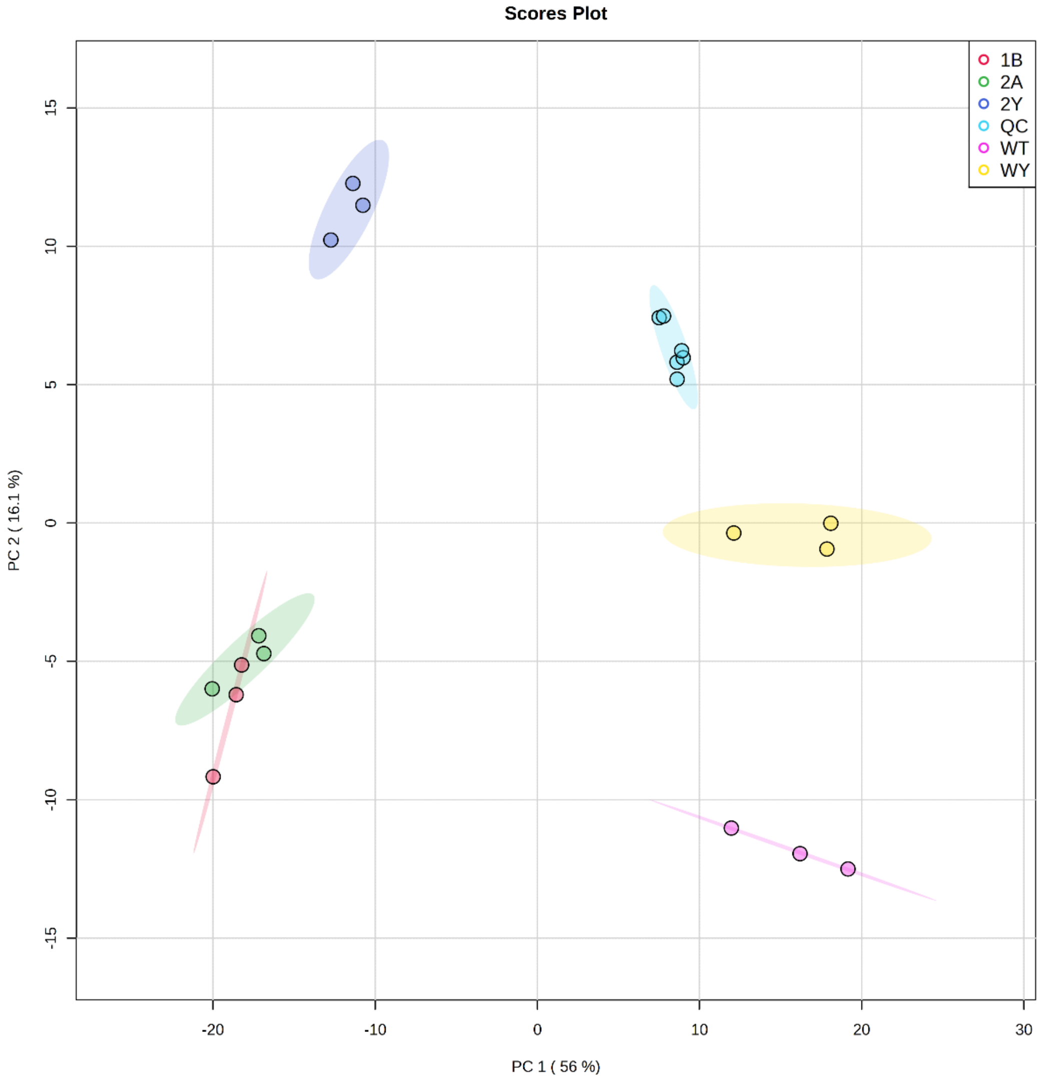

Figure S2. PCA score plot showing the discrimination between each group.

1B: *M. alpina-MaDGAT1B*, 2A: *M. alpina-MaDGAT2A*, 2Y: *M. alpina-MaDGAT2A* with 0.5 g/L linseed oil supplementation, QC: quality control, WT: *M. alpina* ATCC 32222, WY: *M. alpina* ATCC 32222 with 0.5 g/L linseed oil supplementation. For each group, 3 biological replicates were used.

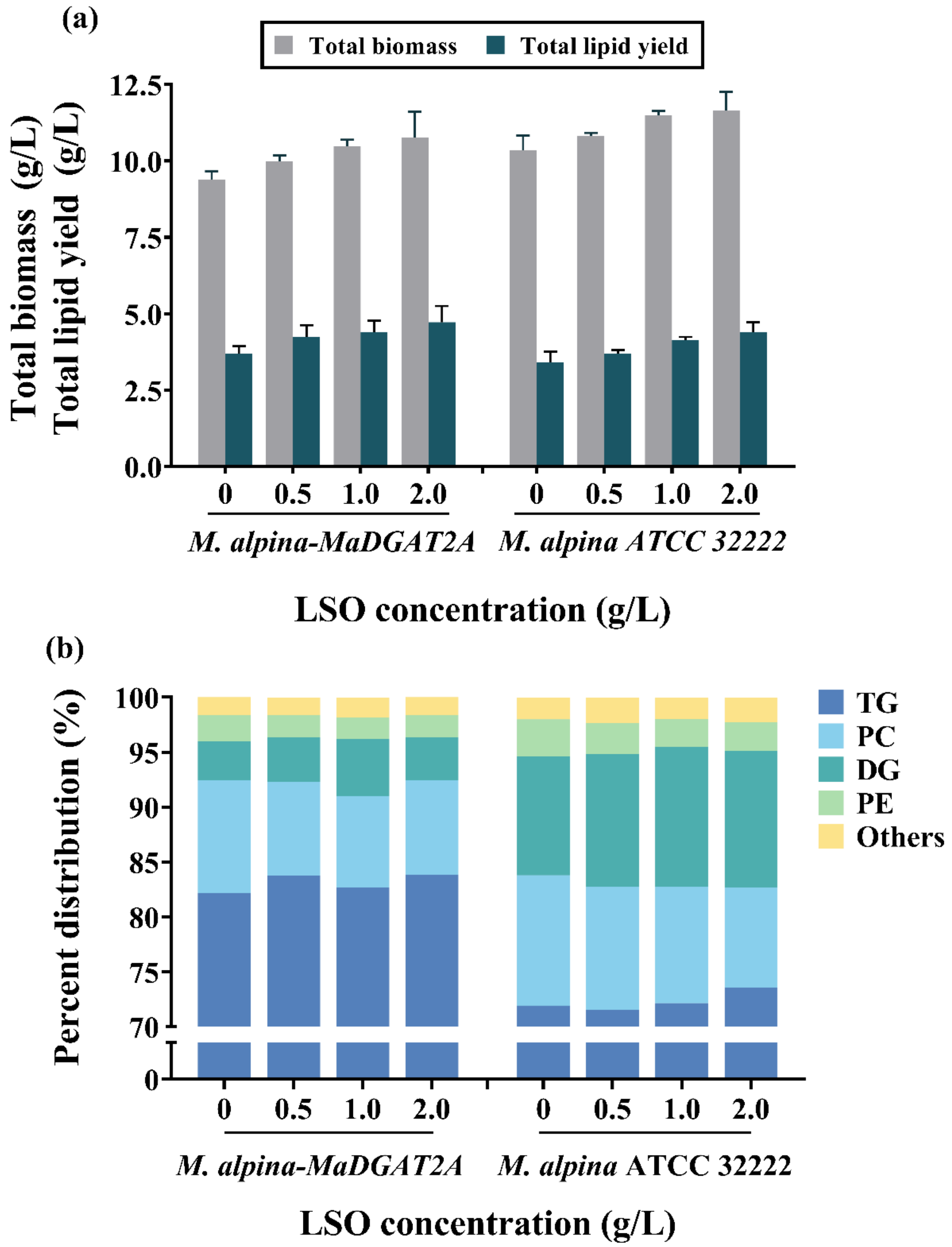

Figure S3. The effect of different concentrations of LSO on *M. alpina-MaDGAT2A* and *M. alpina* ATCC 32222. (a), Total biomass and total lipid yield of *M. alpina-MaDGAT2A* and *M. alpina* ATCC 32222. (b), content of major lipids with different concentrations of LSO in *M. alpina-MaDGAT2A* and *M. alpina* ATCC 32222.

Table S1. Fatty acid profiles of *M. alpina-MaDGAT1B, M. alpina-MaDGAT2A,* and *M. alpina* ATCC 32222. Fatty acids that accounting for more than 1% of total fatty acids were listed.

Fatty acid	content of fatty acids (% of TFA)		
	M. alpina ATCC 32222	*M. alpina-MaDGAT1B*	*M. alpina-MaDGAT2A*
C16:0	11.84±0.25	15.26±0.25	14.64±0.2
C18:0	12.77±0.41	16.14±0.27	16.07±0.17
C18:1	12.23±0.74	15.01±0.18	13.37±1.03
C18:2	7.27±0.34	9.55±0.19	9.22±0.19
C18:3 (GLA)	4.14±0.12	4.48±0.05	4.5±0.11
C20:0	1.36±0.04	1.21±0.03	1.27±0.03
C20:2	0.97±0.07	0.87±0.03	0.86±0.03
C20:3 (DGLA)	3.55±0.19	4.26±0.1	4.49±0.13
C20:4 (ARA)	41.93±1.31	29.66±0.52	32.02±1.2
C22:0	1.82±0.11	1.45±0.1	1.43±0.02
TFA (mg g^{-1} of DCW)	329.56±19.13	402.02±6.17	392.04±16.08

Table S2. Fatty acid profiles of *M. alpina-MaDGAT2A* and *M. alpina* ATCC 32222 with LSO supplementation. Fatty acids that accounting for more than 1% of total fatty acids were listed.

	content of fatty acids (% of TFA)				
	M. alpina-MaDGAT2A		*M. alpina* ATCC 32222		Linseed Oil
Fatty acid / LSO Concentration (g/L)	0.0	0.5	0.0	0.5	
C16:0	14.64±0.2	13.16±0.91	11.84±0.25	11.43±0.18	6.32±0.08
C18:0	16.07±0.17	14.59±1.05	12.77±0.41	12.32±0.34	5.17±0.04
C18:1	13.37±1.03	12.83±0.68	12.23±0.74	12.13±0.93	23.82±0.01
C18:2	9.22±0.19	9.59±0.09	7.27±0.34	7.73±0.22	17.05±0.06
C18:3 (GLA)	4.5±0.11	4.41±0.04	4.14±0.12	3.9±0.03	
C18:3 (ALA)	0.02±0.02	3.89±1.26	0.02±0.01	3.04±0.45	46.44±0.2
C20:0	1.27±0.03	1.18±0.08	1.36±0.04	1.31±0.03	
C20:2	0.86±0.03	0.69±0.09	0.97±0.07	0.98±0.09	
C20:3 (DGLA)	4.49±0.13	4.19±0.05	3.55±0.19	3.27±0.1	
C20:4 (ARA)	32.02±1.2	32.23±1.66	41.93±1.31	40.26±2.11	
C22:0	1.43±0.02	1.37±0.09	1.82±0.11	1.72±0.07	
TFA (mg g^{-1} of MDW)	392.04±16.08	425.57±29.89	329.56±19.13	342.36±6.95	
